# Rsp5 Ubiquitin Ligase Is Required for Protein Trafficking in *Saccharomyces cerevisiae* COPI Mutants

**DOI:** 10.1371/journal.pone.0039582

**Published:** 2012-06-26

**Authors:** Katarzyna Jarmoszewicz, Katarzyna Łukasiak, Howard Riezman, Joanna Kaminska

**Affiliations:** 1 Department of Genetics, Institute of Biochemistry and Biophysics, Polish Academy of Sciences, Warsaw, Poland; 2 Department of Biochemistry, University of Geneva, Geneva, Switzerland; Texas A&M University, United States of America

## Abstract

Retrograde trafficking from the Golgi to the endoplasmic reticulum (ER) depends on the formation of vesicles coated with the multiprotein complex COPI. In *Saccharomyces cerevisiae* ubiquitinated derivatives of several COPI subunits have been identified. The importance of this modification of COPI proteins is unknown. With the exception of the Sec27 protein (β’COP) neither the ubiquitin ligase responsible for ubiquitination of COPI subunits nor the importance of this modification are known. Here we find that the ubiquitin ligase mutation, *rsp5-1*, has a negative effect that is additive with *ret1-1* and *sec28Δ* mutations, in genes encoding α- and ε-COP, respectively. The double *ret1-1 rsp5-1* mutant is also more severely defective in the Golgi-to-ER trafficking compared to the single *ret1-1*, secreting more of the ER chaperone Kar2p, localizing Rer1p mostly to the vacuole, and increasing sensitivity to neomycin. Overexpression of ubiquitin in *ret1-1 rsp5-1* mutant suppresses vacuolar accumulation of Rer1p. We found that the effect of *rsp5* mutation on the Golgi-to-ER trafficking is similar to that of *sla1Δ* mutation in a gene encoding actin cytoskeleton proteins, an Rsp5p substrate. Additionally, Rsp5 and Sla1 proteins were found by co-immunoprecipitation in a complex containing COPI subunits. Together, our results show that Rsp5 ligase plays a role in regulating retrograde Golgi-to-ER trafficking.

## Introduction

The trafficking of proteins between membrane-delimited organelles is mediated by vesicles which form on one membrane and fuse with another. Vesicle formation is mediated by coat proteins that form a lattice on the vesicle surface. One such coat is COPI composed of the Arf1 GTPase and two subcomplexes: F-COPI (β, γ, δ, and ζ subunits) and B-COPI (α, β’, ε) [1]. Individual COPI components interact with cargo proteins through specific signal sequences located in their cytosolic sequences and target them to appropriate transport vesicles. The best described signal sequence is C-terminal K(X)KXX (di-lysine motif) which interacts with subunits of the B-COPI subcomplex; the coatomer isolated from the *sec27-1* (β’-COP) or *ret1-1* (α-COP) yeast mutants fails to bind this signal *in vitro* [1], [2]. *In vitro* cross-linking experiments have also identified γ-COP, a subunit of the F-COPI subcomplex, as the binding partner for the di-lysine motif [1] and γ-COP was also found to bind the cytosolic protein Cdc42 (Rho-related GTPase) [3].

Other proteins, e.g., ER transmembrane proteins, use the receptor protein Rer1 for packing into COPI vesicles. Rer1p interacts with subunits of the COPI coat through its cytoplasmic signals. One of these signals is similar to the di-lysine motif and the other is a tyrosine signal motif [4]. Soluble cargo proteins like the ER chaperone Kar2p, which are unable to interact with the coat, have to use receptors for efficient incorporation into vesicles [1].

In yeast COPI-coated vesicles mediate the retrograde transport from the Golgi apparatus to the endoplasmic reticulum (ER). There is some evidence suggesting an additional function for a subset of COPI subunits in post-Golgi trafficking steps. It has been found in yeast that endocytic cargo, the uracil permease Fur4p or the α factor receptor Ste2p, accumulates on endosomes in some COPI mutants [5]. Also, the transport of biosynthetic cargo, carboxypeptidase S (CPS), is partially blocked in these COPI mutants. Additionally, some COPI mutants are impaired in the recycling of Snc1p, a v-SNARE (vesicle membrane soluble *N*-ethylmaleimide-sensitive factor attachment protein receptor), from endosomes to the Golgi [6].

Interactions of the coat with various proteins may regulate coat specificity on different membranes. This specificity is achieved by posttranslational modifications of coat subunits, e.g., phosphorylation or ubiquitination. A screen for membrane-associated ubiquitinated proteins has identified two of the seven coatomer subunits– α-COP and β’-COP (encoded in yeast by *RET1* and *SEC27*, respectively) [7]. Other studies also found COPI components, such as Ret3p, Sec21p, Sec26p, Sec27p and Sec28p to be ubiquitinated [8]. However the importance of COPI subunit modification with ubiquitin is not well documented.

Attachment of ubiquitin to a protein is a multistep process which requires a ubiquitin activating enzyme, ubiquitin-conjugating enzymes, and ubiquitin-protein ligases. Ubiquitination is also a very diverse modification. Attachment of a single ubiquitin molecule (monoubiquitination) has been shown to control numerous processes such as receptor endocytosis, viral budding and DNA repair [9]–[11]. Attachment of several monoubiquitins to several lysines of a protein is called multiubiquitination. Proteins are also modified with poly-ubiquitin chains in which subsequent ubiquitin molecules are linked C-terminally to a lysine residue in the preceding ubiquitin. When lysine 48 (K48) of ubiquitin is the site of the linkage, such poly-ubiquitination marks the protein for degradation by 26S proteasome [12]. However, poly-ubiquitin chains can also be formed through other lysines present in ubiquitin, K6, K11, K27, K29, K33 or K63, resulting in various conformations of the ubiquitin chains and as a consequence a range of molecular signals [13]. Ubiquitination is reversible - ubiquitins can be removed by specific deubiquitinating enzymes - ubiquitin proteases (Ubps) and ubiquitin C-terminal hydrolases (Uch) [14].

In the yeast *Sacharomyces cerevisiae* the ubiquitin ligase Rsp5p has been shown to tag proteins with monoubiquitin or with chains formed through K63 [15]. The Rsp5-dependent modification is important for several processes including inheritance of mitochondria, chromatin remodelling, and activation of transcription factors. The role of Rsp5 ligase in the endocytosis of several plasma membrane transporters, channels and permeases and intracellular trafficking of proteins has also been documented thoroughly [16]. Rsp5p participates also in the sorting of permeases like Fur4p or the general amino acid permease, Gap1p, at Golgi apparatus and in the sorting of several cargoes in multivesicular bodies (MVB) [16]. This action of Rsp5p at several distinct locations is believed to be achieved by interactions with different adaptor proteins. These adaptors are also required for ubiquitination of those Rsp5p substrates that lack motifs for Rsp5p binding. Such adaptors have been described for endocytic cargoes and for the sorting at the Golgi. Rsp5p can also affect intracellular transport by influence on actin cytoskeleton organization. Rsp5p has several substrates among actin-cytoskeleton proteins. The described *in vivo* and *in vitro* substrates for Rsp5 are Sla1, Lsb1, Lsb2 - proteins that bind to Las17 (an activator of Arp2/3 complex required for actin polimerization), Rvs167 - a protein required for viability upon starvation and Sla2 [17]. In the case of Sla1 protein Rsp5-dependent ubiquitination causes its processing [18] but the physiological role of ubiquitination of most of actin cytoskeleton proteins is unknown.

Genetic and biochemical evidence indicates that the deubiquitinating enzyme Ubp2p antagonizes Rsp5p activity [19]. In contrast, a lack of Ubp3p activity (*ubp3Δ* mutation) seems to have an additive negative effect on the growth of an *rsp5* mutant – a double *ubp3Δ rsp5* mutant shows synthetic growth defect [20]. Moreover, Rsp5p cooperates with Ubp3p in the regulation of ribophagy, a specific type of autophagy responsible for degradation of ribosomes [20]. Recently Rsp5p was shown *in vitro* to ubiquitinate Sec23p, a subunit of COPII coat [21] and Ubp3p is responsible for Sec23p deubiquitination [22]. Ubp3p and its cofactor Bre5p were also shown to be responsible for deubiquitination of Sec27p (β’COP). Modulation of Sec27p ubiquitination status has a regulatory role. Only after Ubp3-catalyzed deubiquitination is Sec27p able efficiently to bind cargo containing the di-lysine motif [22].

Here we asked if ubiquitin ligase Rsp5p, together with the Ubp3p-Bre5p complex, regulates Golgi-to-ER retrograde trafficking. We found that a lack of the Bre5p cofactor (*bre5Δ*) combined with the *rsp5-1* mutation has an additive negative effect on yeast growth and on the trafficking of GFP-Rer1 marker fusion protein. We also show that Rsp5p is necessary when COPI function is impaired due to *ret1-1* mutation in the gene encoding its α subunit. The *rsp5-1* mutation combined with *ret1-1* compromises Golgi-to-ER trafficking as evidenced by vacuolar localization of the Rer1p receptor and secretion of Kar2p (an ER chaperone). Overexpression of ubiquitin was able to suppress the vacuolar accumulation of GFP-Rer1 in the *ret1-1 rsp5-1* mutant. However, ubiquitinated forms of Sec27p were still found in the *rsp5-1* mutant suggesting the action of another ubiquitin ligase(s). Further genetic studies suggest that Rsp5p influences Golgi-to-ER trafficking by regulating the actin cytoskeleton. Taken together, our results show a new role of Rsp5p ligase together with Bre5p and actin cytoskeleton in the regulation of trafficking from the Golgi to the ER.

## Results

### *bre5Δ* and *rsp5* Mutations have Additive Effect on Trafficking from Golgi to ER

**Figure 1:**
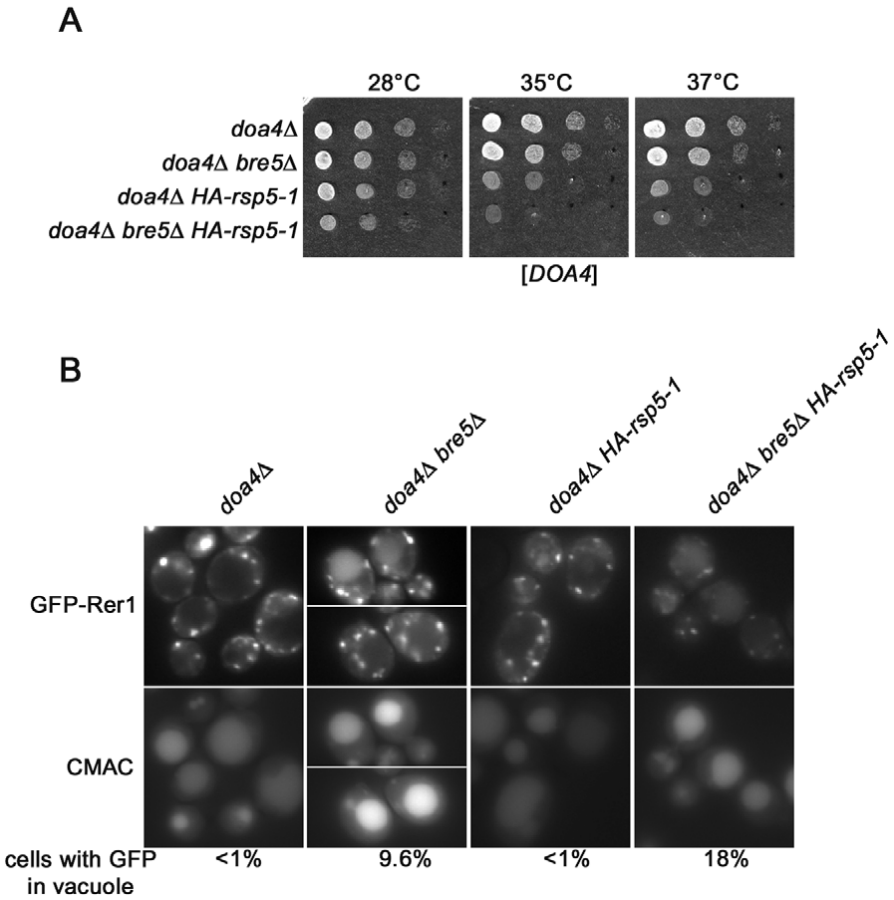
*rsp5* and *bre5* mutations have additive effect on growth and mislocalization of GFP-Rer1. (**A**) *bre5Δ* and *HA-rsp5-1* mutations show weak genetic interaction. Strains *doa4Δ, doa4Δ bre5Δ, doa4Δ HA-rsp5-1* and *doa4Δ bre5Δ HA-rsp5-1* were transformed with plasmid encoding *DOA4*. Serial 1∶10 dilutions of transformants were spotted on YPD and incubated at indicated temperatures. (**B**) *doa4Δ bre5Δ HA-rsp5-1* mutant accumulates GFP-Rer1 in vacuole. Plasmid encoding GFP-Rer1 fusion was transformed into same mutants as in panel A. Transformants were grown on SC -ura at 28°C and GFP-Rer1 was observed by fluorescence (GFP). Cells were stained with CMAC to visualize vacuole. Percentage of cells accumulating GFP in vacuole is given.

It has been reported that the double *rsp5 ubp3Δ* mutant is inviable at a temperature permissive for the single *rsp5* and *ubp3Δ* mutants and the *rsp5* and *ubp3Δ* mutations have an additive effect on ribophagy [20]. Additionally, Ubp3p, together with its cofactor Bre5p, is involved in deubiquitination of Sec23p, a subunit of COPII coat. Recently it was shown that Sec23p is ubiquitinated by Rsp5p [21]. The single *ubp3Δ* and *bre5Δ* mutants were shown to accumulate ubiquitinated Sec27p, a β’-COPI subunit [22]. Therefore, we asked if Rsp5p also acts with the Ubp3p-Bre5p complex in retrograde Golgi-to-ER trafficking. First we tested the genetic interaction between *rsp5-1* mutation, which carries an amino acid substitution in the catalytic Hect domain, and deletion of the *BRE5* gene (*bre5Δ*) in a *doa4Δ* background (see below for explanation). A comparison of growth of strains *doa4Δ bre5Δ, doa4Δ HA-rsp5-1* and *doa4Δ bre5Δ HA-rsp5-1* on YPD at different temperatures revealed that the *doa4Δ bre5Δ HA-rsp5-1* mutant grew worse at 35°C than did the *doa4Δ bre5Δ* or *doa4Δ HA-rsp5-1* mutants, indicating a very weak genetic interaction between *bre5Δ* and *rsp5-1* (not shown). The same genetic interaction was visible when all above strains were transformed with a plasmid bearing the *DOA4* gene (Figure 1A). This interaction is in agreement with data from a genetic interaction map [23].

**Figure 2:**
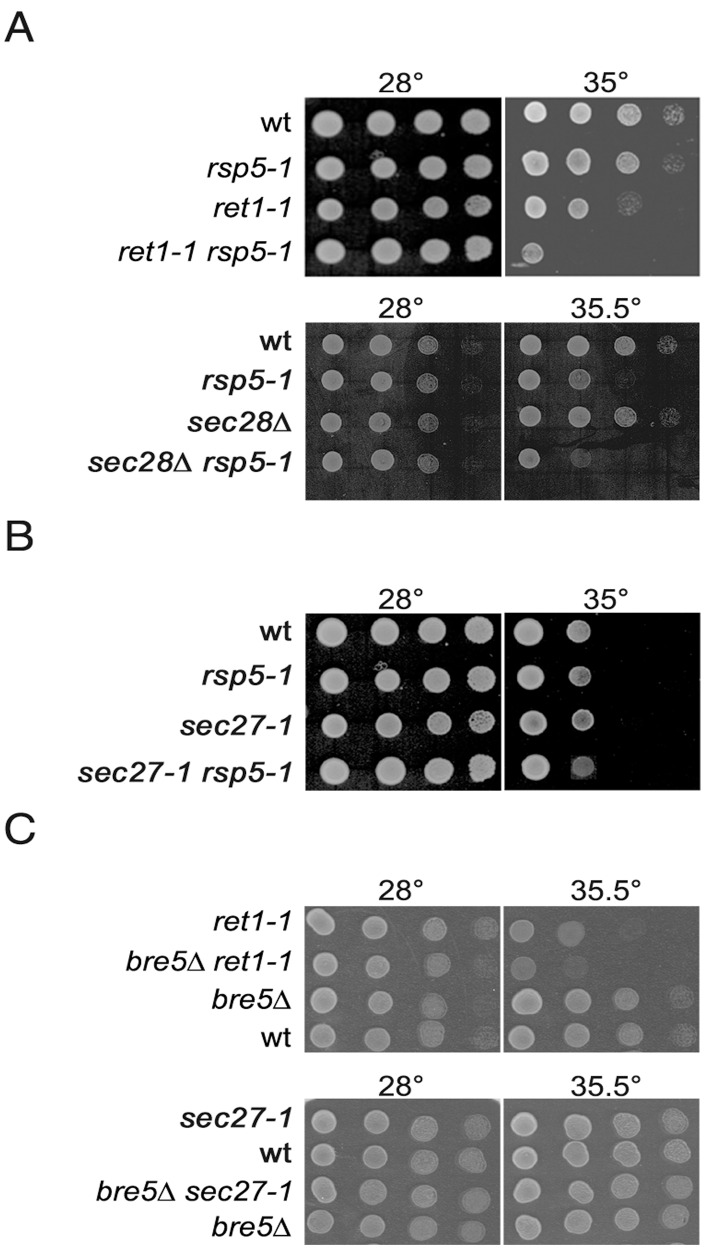
Genetic interaction between *rsp5-1* or *bre5Δ* and mutations in genes encoding COPI subunits. (**A**) Negative genetic interaction between *rsp5-1* and *ret1-1* or *sec28Δ* mutations. (**B**) No genetic interaction between *rsp5-1* and *sec27-1* mutation. (**C**) Negative genetic interaction between *bre5Δ* and *ret1-1* and no genetic interaction between *bre5Δ* and *sec27-1*. Serial 1∶10 dilutions of spore clones from crosses *ret1-1* × *rsp5-1* (RH3042 × FW1808), *sec28Δ* × *rsp5-1* (KJK39 × FW1808), *sec27-1* × *rsp5-1* (RH359-7D × FW1808), *bre5Δ* × *ret1-1* (JK140-5A × JK82-4B) and *bre5Δ* × *sec27-1* (JK140-5A × JK84-3C) were spotted on YPD medium and incubated for 2 or 3 days at indicated temperatures.

Next we asked if genetic interaction between *bre5Δ* and *rsp5-1* was reflected in compromised Golgi-to-ER trafficking. To probe the latter we used GFP-tagged Rer1p. Rer1p is a Golgi membrane protein that is required for the retrieval of escaped ER membrane proteins back from the Golgi [4]. Vacuolar accumulation of Rer1p is characteristic for mutants defective in the retrograde trafficking from Golgi to ER [4]. As shown in Figure 1B, in *doa4Δ* cells GFP-Rer1p exhibits a Golgi pattern of fluorescence typical for wild type yeast. In about 10% of the *doa4Δ bre5Δ* cells the GFP-Rer1 signal overlapped with vacuole staining by the vacuolar marker, blue CMAC, suggesting vacuolar accumulation of Rer1. This confirms that the *bre5Δ* mutant is defective in the Golgi-to-ER retrograde trafficking. In contrast, the *doa4Δ HA-rsp5-1* mutant showed localization of GFP-Rer1 similar to that of *doa4Δ* cells. In about 18% of the *doa4Δ bre5Δ HA-rsp5-1* mutant cells the GFP-Rer1 fusion protein was found in vacuoles. Additional punctuate and diffuse GFP fluorescence was also observed in those cells (Figure 1B). This suggests that the defect in retrograde trafficking in the *doa4Δ bre5Δ HA-rsp5-1* mutant is slightly stronger than in *doa4Δ bre5Δ*. The more prevalent diffuse distribution of GFP-Rer1 in the triple mutant indicates that the delivery of GFP-Rer1 to the vacuole at trans-Golgi or endosomes may also be partially defective.

The above data show that Rsp5p ligase acts together with Bre5p in retrograde Golgi-to-ER trafficking.

**Figure 3:**
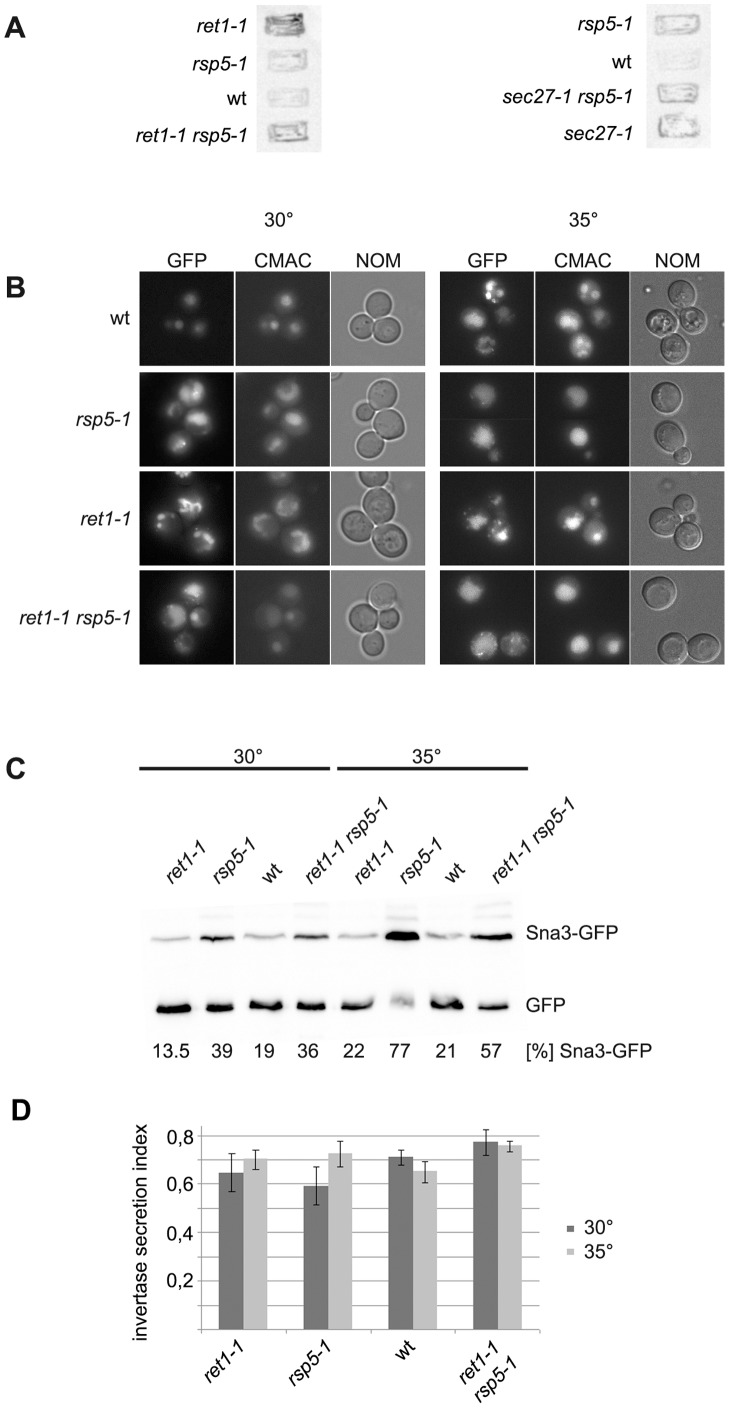
CPY secretion and Sna3-GFP trafficking and invertase activity are the same in *ret1-1 rsp5-1* mutant and in single *rsp5-1*. (**A**) Double mutants *ret1-1 rsp5-1* and *sec27-1 rsp5-1* do not secrete more CPY compared with the single *ret1-1*, *sec27-1* or *rsp5-1* mutants. Spore clones (as in Figure 2) were replica-plated onto nitrocellulose filters and grown on solid YPD for 1 day at 28°C. Cells secreting CPY were identified by Western blotting with anti-CPY antibody. (**B**) Sna3-GFP trafficking defect caused by *rsp5-1* mutation is not augmented by *ret1-1*. Plasmid encoding Sna3-GFP was transformed into spore clones as in Figure 2A. Transformants were grown to mid logarithmic phase on SC -ura at 30°C or shifted to 35°C for 1 hour. Sna3-GFP was observed by fluorescence (GFP). Cells were stained with CMAC to visualize vacuole and viewed with Nomarski optics (NOM). (**C**) Whole cell lysates form transformants from Figure 3B were analyzed by Western blotting with anti-GFP antibody. Percentage of Sna3-GFP in total GFP signal (GFP and Sna3-GFP) is given. (**D**) Invertase activity was assayed in spore clones as in Figure 2A. Cultures were grown to mid logarithmic phase at 30°C and shifted or not to 35°C for 30 minutes. The proportion between activity of secreted invertase to the total invertase activity (invertase secretion index) is shown.

### The *rsp5-1* Mutation Shows Genetic Interaction with *ret1-1* and *sec28Δ*

To better understand the role of Rsp5p ligase in retrograde trafficking we tested for genetic interaction between the *rsp5-1* mutation and mutations in genes encoding COPI subunits: *sec27-1*, *sec28Δ* and *ret1-1*. Growth of spores from crosses *rsp5-1* × *ret1-1*, *rsp5-1* × *sec27-1*, and *rsp5-1* × *sec28Δ* was tested and the phenotypes of the respective double mutants were scored. As shown in Figure 2A, the double *ret1-1 rsp5-1* mutant stops growing at 35°C, a temperature permissive for the single *ret1-1* and *rsp5-1* mutants. This genetic interaction was not-allele specific. Other *rsp5* alleles such as *rsp5-w1*, *rsp5-w2*, *rsp5-w3* [24] and *rsp5-19* [25] with mutations in the WW domains responsible for Rsp5p interaction with other proteins, also had additive negative effects on *ret1-1* growth (not shown). A negative genetic interaction was also observed between *sec28Δ* and *rsp5-1*. The double *sec28Δ rsp5-1* mutant failed to grow at 35.5°C (Figure 2A). Interestingly, the *sec27-1 rsp5-1* mutant grew as well as did the single *sec27-1* mutant at all temperatures tested (Figure 2B). Thus, various *rsp5* mutations show a genetic interaction with mutated alleles of some genes encoding COPI subunits.

**Figure 4:**
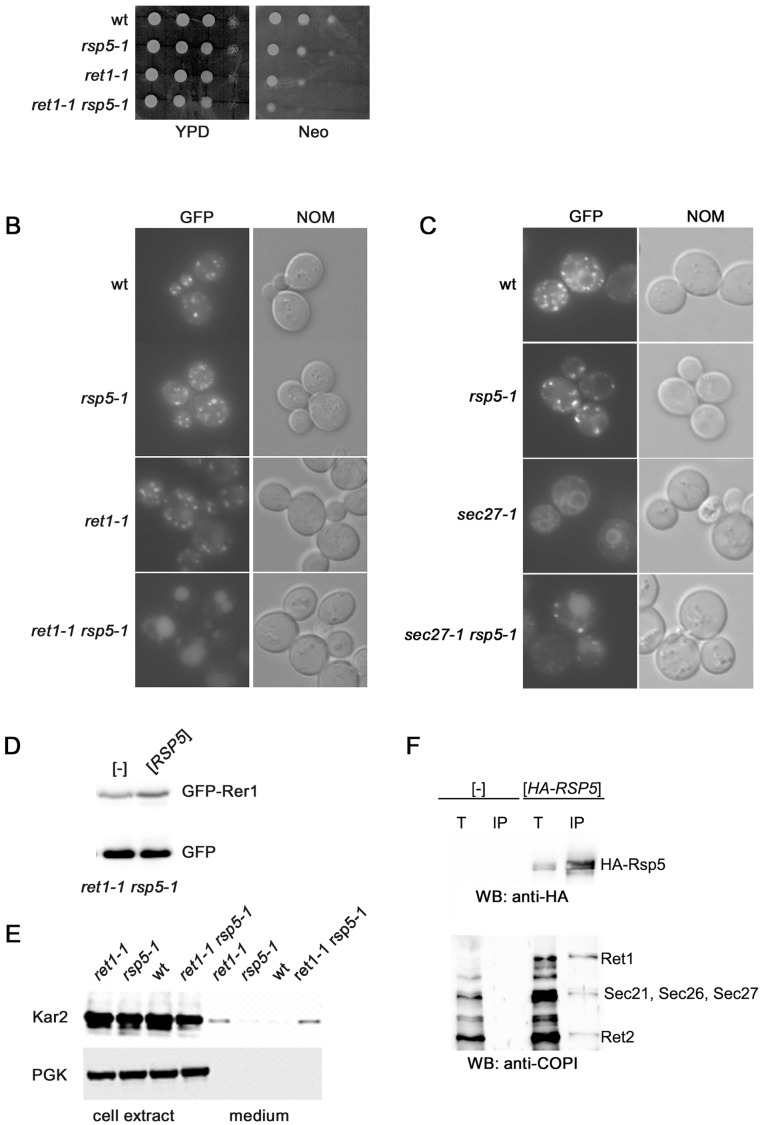
Retrograde trafficking from Golgi to ER is impaired in double *ret1-1 rsp5-1* mutant. (**A**) Double mutant *ret1-1 rsp5-1* is sensitive to neomycin. Spore clones from *ret1-1* × *rsp5-1* cross (as in Figure 2) were serially diluted 1∶10, spotted on YPD or YPD containing 1 mM neomycin (Neo) and grown for 1 day at 28°C. (**B** and **C**) Defect of GFP-Rer1 trafficking in *ret1-1 rsp5-1* and *sec27-1 rsp5-1* mutants. Spore clones (as in Figure 2 and 3B) from *ret1-1* × *rsp5-1* and *sec27-1* × *rsp5-1* crosses were transformed with plasmid encoding *GFP-RER1*. Transformants were grown on SC -ura at 28°C. GFP-Rer1 was localized by fluorescence (GFP) and cells were viewed with Nomarski optics (NOM). (**D**) *rsp5-1* mutation is responsible for additional defect in GFP-Rer1 trafficking caused by *ret1-1*. Centromeric vector encoding *HA-RSP5* or empty vector ([-]) were transformed into *ret1-1 rsp5-1* expressing *GFP-RER1*. Whole cell protein extracts from transformants were analysed by Western blotting with anti-GFP antibody. (**E**) Secretion of Kar2p is enhanced in *ret1-1 rsp5-1* mutant. Spore clones from *ret1-1* × *rsp5-1* cross (as in B) were grown at 28°C in YPD, transferred to fresh medium and incubated at 28°C for 1 h. Whole cell protein extracts and proteins TCA-precipitated from medium were analyzed by Western blotting with anti-Kar2 and anti-PGK antibody. The latter was to control cell integrity. (**F**) HA-Rsp5 binds COPI complex. The extracts from *rsp5Δ* strain transformed with empty vector ([-]) or with centromeric plasmid YCpHA-RSP5 ([*HA-RSP5*]) were used for immunoprecipitation using anti-HA antibody (16B12). Total extracts (T) and immunoprecipitated fraction (IP) were analysed by Western blotting with anti-HA and with anti-coatomer antibody.

A negative genetic interaction between deletion of the *BRE5* gene and *ret1-1* or *sec27-1* mutations was previously described [26], [27]. However, we observed only an interaction between *bre5Δ* and *ret1-1* and no such interaction between *bre5Δ* and *sec27-1* (Figure 2C). Thus, the *rsp5-1* and *bre5Δ* mutations displayed the same genetic interaction spectrum. This fact together with the additive effects of *rsp5-1* and *bre5Δ* on retrograde trafficking shows that both ubiquitination and deubiquitination processes are important for COPI-dependent trafficking.

**Figure 5:**
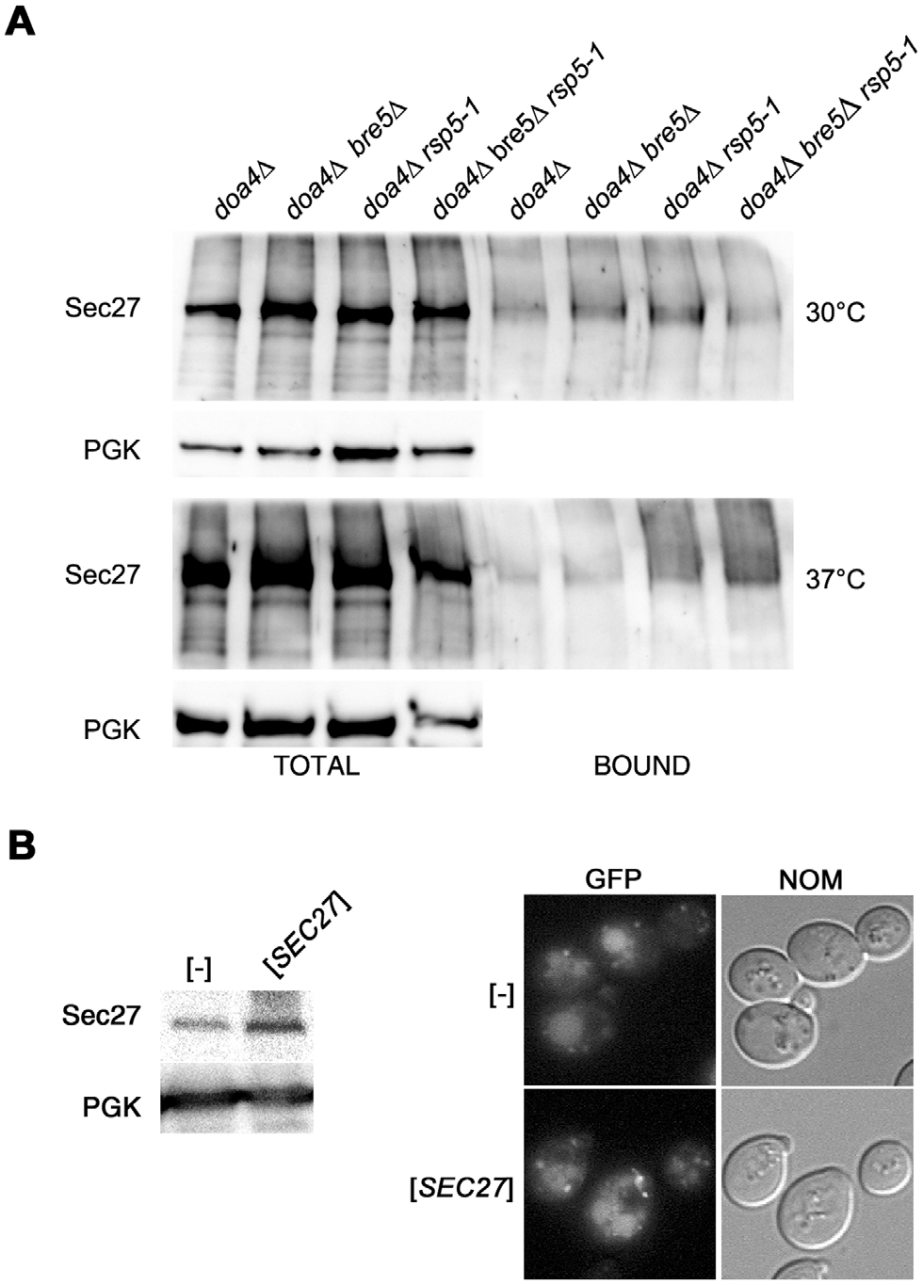
*rsp5-1* mutation does not abolish accumulation of polyubiquitinated Sec27p. (**A**) Mutants *doa4Δ, doa4Δ bre5Δ, doa4Δ HA-rsp5-1,* and *doa4Δ bre5Δ HA-rsp5-1* were transformed with plasmid expressing *His_6_-UBI*. Transfromants were grown to mid-logarithmic phase at 30°C and shifted or not to 35°C for 4 hours. His_6_-Ubi was pulled down on Ni-NTA beads. Total fraction and fraction bound to beads were analysed by Western blotting with anti-Sec27 or with anti-PGK antibody for control. (**B**) *SEC27* is overproduced. Multicopy vector encoding *SEC27* or empty vector ([-]) were transformed into *ret1-1 rsp5-1* strain expressing *GFP-RER1*. Whole cell protein extracts from transformants were analysed by Western blotting with anti-Sec27 or with anti-PGK antibody for a control. (**C**) Overexpression of *SEC27* causes fragmentation of vacuoles. The same transformants as in A were grown on SC -ura -trp at 28°C. GFP-Rer1 was localized by fluorescence (GFP) and cells were viewed with Nomarski optics (NOM).

### Defect in Golgi-to-ER Trafficking of Cargo Proteins in *ret1-1 rsp5-1* Mutant

Rsp5 participates in the sorting of several cargoes in multivesicular bodies (MVB). It is also known that in some COPI mutants the transport of a biosynthetic cargo sorted via the MVB, carboxypeptidase S (CPS), is partially blocked at a post-Golgi step [5]. So we first investigated if the observed genetic interaction between *rsp5-1* and *ret1-1* could be due to an additive effect on cargo sorting in endosomes affecting MVB formation. To test this possibility the trafficking of the vacuolar carboxypeptidase Y (CPY) was monitored. In mutants defective in MVB formation CPY is released to the extracellular space. The *sec27-1* mutant was characterized as secreting more CPY compared to wild type cells [5]. The level of CPY secretion by spore clones from the *ret1-1* × *rsp5-1* and *sec27-1* × *rsp5-1* crosses was monitored, but neither of the double mutants showed enhanced secretion of CPY compared with the single *ret1-1*, *sec27-1* or *rsp5-1* mutants (Figure 3A). Another type of cargo known to require Rsp5p for proper segregation into the lumen of MVB is Sna3p. Sna3p binds to Rsp5p and its sorting into MVB is affected by *rsp5-1* mutation [28]. The localization of Sna3-GFP and the level of free GFP released from Sna3-GFP in vacuole lumen were monitored in spore clones from the *ret1-1* × *rsp5-1* cross. As shown in Figure 3B in the wild type, *ret1-1*, *rsp5-1* and *ret1-1 rsp5-1* strains Sna3-GFP was present in the vacuole. However, in *rsp5-1* and *ret1-1 rsp5-1* more intact Sna3-GFP was present (39% and 36% respectively) than in *ret1-1* and wild type cells (13.5% and 19%) as assessed by western blotting with anti-GFP antibody (Figure 3C). After a shift to 35°C for 2 hours, a temperature restrictive for the *ret1-1 rsp5-1* mutant, more Sna3-GFP was present in cytoplasmic foci in *rsp5-1* and *ret1-1 rsp5-1* mutants. Western blotting analysis revealed that in these strains, respectively, 77% and 57% of total GFP signal comes from the intact Sna3-GFP fusion (Figure 3C). This result shows that addition of the *ret1-1* mutation to *rsp5-1* does not cause a stronger defect in Sna3-GFP transport. From these experiments we conclude that the observed genetic interaction between the *ret1-1* and *rsp5-1* mutations is probably not due to an additive negative effect on the sorting into the MVB. Rsp5p ligase is apparently necessary when COPI is defective but at a trafficking step other than MVB sorting. Recently the ubiquitination of Sec23p, a subunit of COPII coat has been shown to be mediated by Rsp5p [21]. Thus, the genetic interaction between *ret1-1* and *rsp5-1* could be a result of their additive effect on the anterograde transport between ER and Golgi. Alternatively, Rsp5 could regulate Golgi-to-ER transport. The first possibility was ruled out. We tested the activity of extracellular invertase. As shown in Figure 3D neither single *ret1-1* or *rsp5-1* mutant nor the double *ret1-1 rsp5-1* had lowered activity of extracellular invertase at 30°C nor after a shift to 35°C, a temperature restrictive for double *ret1-1 rsp5-1* mutant.

The possibility that Rsp5p influences Golgi-to-ER trafficking was verified by monitoring the transport of three different types of cargo in the *rsp5-1* mutant. To study the trafficking of proteins containing the di-lysine motif we tested sensitivity to neomycin, an aminoglycoside antibiotic which competes with the di-lysine motif for coatomer binding [29]. We assumed that when the interaction of the cargo with COPI is impaired, but not abolished, due to the *ret1-1* mutation, addition of neomycin should cause an additional defect in retrograde trafficking and thus should be deleterious to the *ret1-1* mutant. Indeed, as shown in Figure 4A, the *ret1-1* mutant is more sensitive to neomycin than the wild type. We also tested if the *rsp5-1* mutation further intensified the neomycin sensitivity caused by the *ret1-1* mutation. We found that the *rsp5-1* mutation had an additive effect to that caused by the *ret1-1* mutation. In contrast there was no additive effect on the growth on neomycin-containing media between *sec27-1* and *rsp5-1* (not shown). Because neomycin interferes with other processes in the cell besides affecting coatomer we cannot exclude other reasons of the observed effect of *rsp5-1* on neomycin sensitivity. However, these results are at least in agreement with the idea that Rsp5p ligase is needed for proper di-lysine motif interaction with the COPI complex.

The Rer1-dependent retrograde trafficking was monitored by observing GFP-Rer1 localization. There was no defect in GFP-Rer1 localization in the *rsp5-1* mutant even after a shift to 37°C (Figure 1B, 4B, 4C and not shown). Thus, the *rsp5-1* mutation alone does not block retrograde trafficking. We then checked if *rsp5-1* enhances the defect in retrograde trafficking caused by the *ret1-1* or *sec27-1* mutations. A vacuolar localization of GFP-Rer1 had already been described for the single *ret1-1* mutant [4]. In our strain this was visible only in a minor fraction of cells. In contrast, almost all double *rsp5-1 ret1-1* mutant cells had the GFP-Rer1 fusion protein localized to vacuoles even at 28°C, a temperature permissive for grow of this mutant (Figure 4D). In principle, this enhanced vacuolar accumulation of GFP-Rer1 in the *ret1-1 rsp5-1* strain could be a consequence of the *rsp5-1* mutation or of the genetic background of the double mutant. To distinguish between these two possibilities and to quantify the effect of Rsp5p on the trafficking, a centromeric vector encoding *HA-RSP5* was transformed into *ret1-1 rsp5-1* expressing *GFP-RER1*; as a control empty vector was used. Protein extracts were obtained from the transformants and analyzed by Western blotting with anti-GFP antibody. The ratio of free GFP, liberated from the GFP-Rer1 fusion in the vacuole, to that of total GFP (free GFP and GFP-Rer1) was calculated. We found that there is ca. 15% more free GFP in the *ret1-1 rsp5-1* mutant compared to *ret1-1* (Figure 4D). So, the *rsp5-1* mutation has an additive effect to the *ret1-1* mutation on the trafficking of the Rer1p cargo receptor. Interestingly, the double mutant *rsp5-1 sec27-1* also accumulated GFP-Rer1 fusion in the vacuole (Figure 4C).

Next, we analyzed the trafficking of Kar2p. Kar2p is an ER-resident protein which, when misaddressed to the Golgi, interacts with its receptor Erd2p and returns to the ER in a COPI-dependent manner. Blocking the retrograde Golgi-to-ER trafficking causes Kar2p secretion to the medium. We found that the percentage of total Kar2p that is secreted is enhanced in the double *ret1-1 rsp5-1* mutant compared with the single mutants and wild type cells (Figure 4E). No such effect was observed in the *sec27-1 rsp5-1* mutant (not shown). Thus, the effect of the double *ret1-1 rsp5-1* mutation on Kar2p secretion corroborates the genetic interaction of the two mutations described above.

To answer a question how Rsp5 regulates trafficking from the Golgi to ER the binding of Rsp5 to coatomer was tested. For this purpose the *rsp5Δ* strain was transformed with empty vector or vector bearing tagged *RSP5* (*HA-RSP5*). When HA-tagged Rsp5 was immunoprecipitated from cells and analyzed by immunoblot, we observed the presence of COPI subunits together with HA-Rsp5. There were no COPI proteins in the control immunoprecipitation from *rsp5Δ* cells transformed with empty vector (Figure 4F).

We conclude that Rsp5 is found in a complex together with the COPI coat and Rsp5p ligase is necessary for trafficking of different cargo types from the Golgi to the ER when this transport is defective due to mutations in genes encoding COPI subunits.

**Figure 6:**
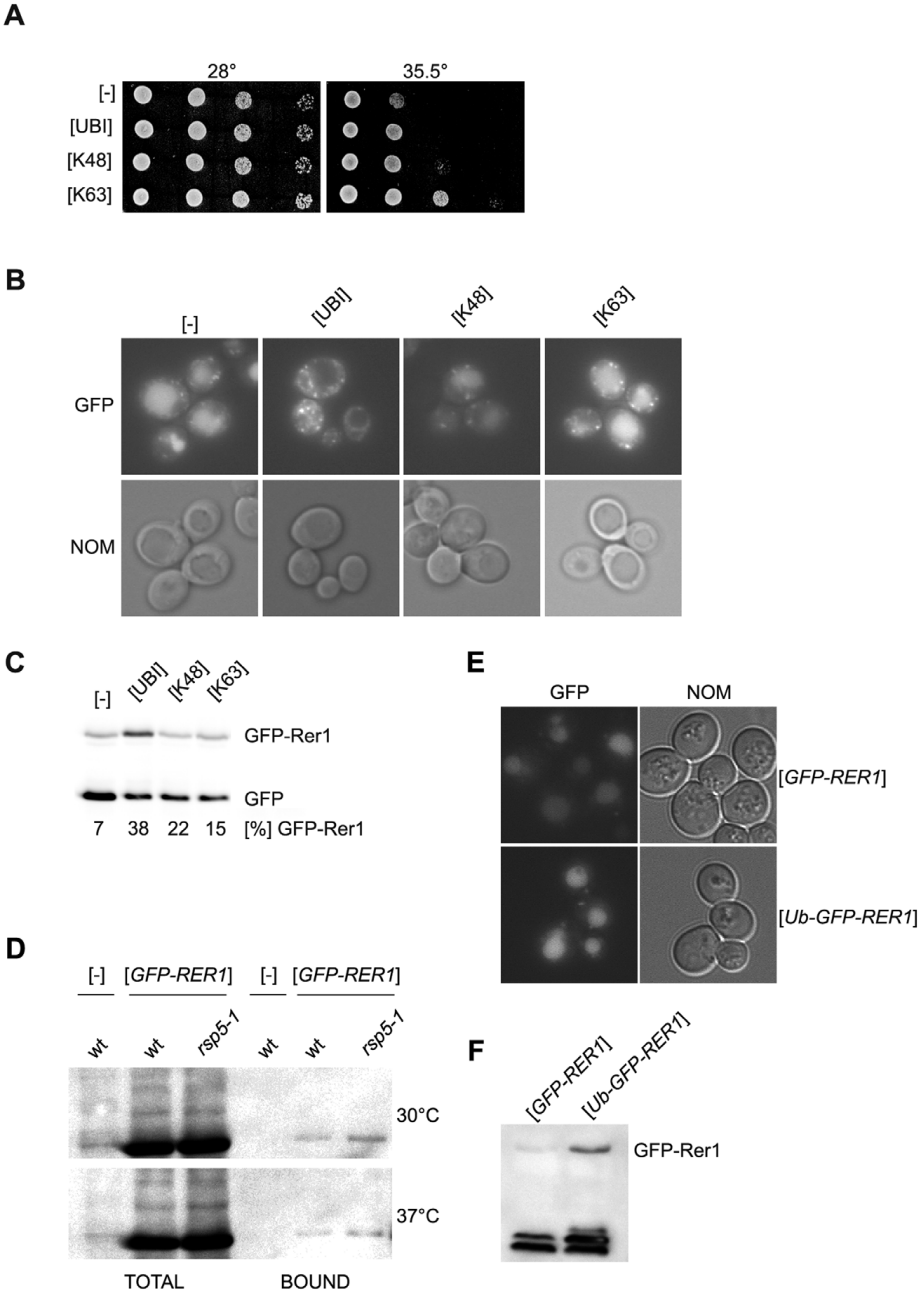
Overexpression of ubiquitin suppresses *ret1-1 rsp5-1* mutant defects. (**A**) Growth defect of *ret1-1 rsp5-1* mutant is suppressed by overexpression of ubiquitin or its variants. *ret1-1 rsp5-1* mutant was transformed with empty vector [-] or with plasmids encoding wild type ubiquitin [UBI], ubiquitin with only single lysine 48 [K48] or 63 [K63] present and all other lysines replaced with arginine. Serial 1∶10 dilutions of transformants were spotted on YPD medium and incubated for 2 days at indicated temperatures. (**B**) Localization of GFP-Rer1 to vacuole in *ret1-1 rsp5-1* mutant is suppressed by overexpression of ubiquitin. Transformants from Figure 6A were additionally transformed with plasmid encoding GFP-Rer1 and were grown on SC -ura -leu at 28°C. Expression of ubiquitin variants was induced by addition of 100 µM CuSO_4_ for 2 hours before observations. GFP-Rer1 was localized by fluorescence (GFP) and cells were viewed with Nomarski optics (NOM). (**C**) Whole cell lysates form transformants from Figure 6B were analyzed by Western blotting with anti-GFP antibody. Percentage of GFP-Rer1 in total GFP signal (GFP and GFP-Rer1) in each lane is given. (**D**) Wild type or *rsp5-1* mutant were transformed with a plasmid expressing *His_6_-UBI* and with empty vector [-] or a plasmid encoding GFP-Rer1. Transfromants were grown to mid-logarithmic phase at 30°C. Expression of ubiquitin was induced by addition of 100 µM CuSO_4_ for 2 hours before cultures were shifted or not to 37°C for 1 hour. His_6_-Ubi was pulled down on Ni-NTA beads. Total fraction and fraction bound to beads were analysed by Western blotting with anti-GFP antibody. (**E**) Fusion protein Ub-GFP-Rer1 is targeted to vacuole. The *rer1-1 rsp5-1* strain was transformed with plasmid encoding one of the fusions *GFP-RER1* or *Ub-GFP-RER1*. The GFP-Rer1 and Ub-GFP-Rer1 proteins were localized by fluorescence (GFP) and cells were viewed with Nomarski optics (NOM). (**F**) Ub-GFP-Rer1 protein is expressed. Total protein extracts from the same transformants as in Figure 6E were analyzed by Western blotting with anti-GFP antibody.

### Sec27 Protein is Ubiquitinated in *rsp5-1* Mutant

Finding that Rsp5 ligase and COPI proteins could be co-immunoprecipitated suggests that Rsp5 ligase might be responsible for ubiquitination of some COPI subunits. Sec27p had been found to be deubiquitinated by the Ubp3p-Bre5p enzyme, which prompted the question whether the observed effect of the *rsp5* mutation on retrograde trafficking could be a result of Rsp5-dependent ubiquitination of Sec27p. To test this we used four strains: *doa4Δ*, *doa4Δ bre5Δ*, *doa4Δ HA-rsp5-1*, and *doa4Δ bre5Δ HA-rsp5-1*. In these strains the *DOA4* gene, which encodes one of the deubiquitinating enzymes, was deleted. The *doa4Δ* mutation decreases the level of free ubiquitin, which can be suppressed by ectopically expressed tagged version of ubiquitin. This allows easier detection of proteins modified with ubiquitin. The *bre5Δ* mutation was introduced to prevent deubiquitination in order to facilitate detection of ubiquitinated Sec27. Strains were transformed with a plasmid expressing His-tagged ubiquitin from the *CUP1* promoter. Cells were grown to mid-exponential phase and half of the culture was shifted to 37°C for 4 hours. Next, protein extracts were prepared from cultures grown at both temperatures and His_6_-Ubi was pulled down on Ni-NTA beads. The total protein extracts and the material bound to the beads were analyzed by Western blotting with anti-Sec27 antibody. As shown in Figure 5A, at 30°C in all strains tested Sec27p was present in the bound fraction. When extracts were made from cultures incubated at 37°C the Sec27p protein was also recovered in all of the strains but accumulation of slower-migrating forms of Sec27p was visible in *doa4Δ HA-rsp5-1*, and *doa4Δ bre5Δ HA-rsp5-1* mutants (Figure 5A). This result can be interpreted in several ways: Sec27p is not ubiquitinated by Rsp5p, is ubiquitinated by Rsp5p and another ligase, or overexpression of ubiquitin suppresses the defect of *rsp5-1* mutation. Indeed ubiquitin is a multicopy suppressor of the temperature sensitivity of the *rsp5-1* mutant [30].

**Figure 7:**
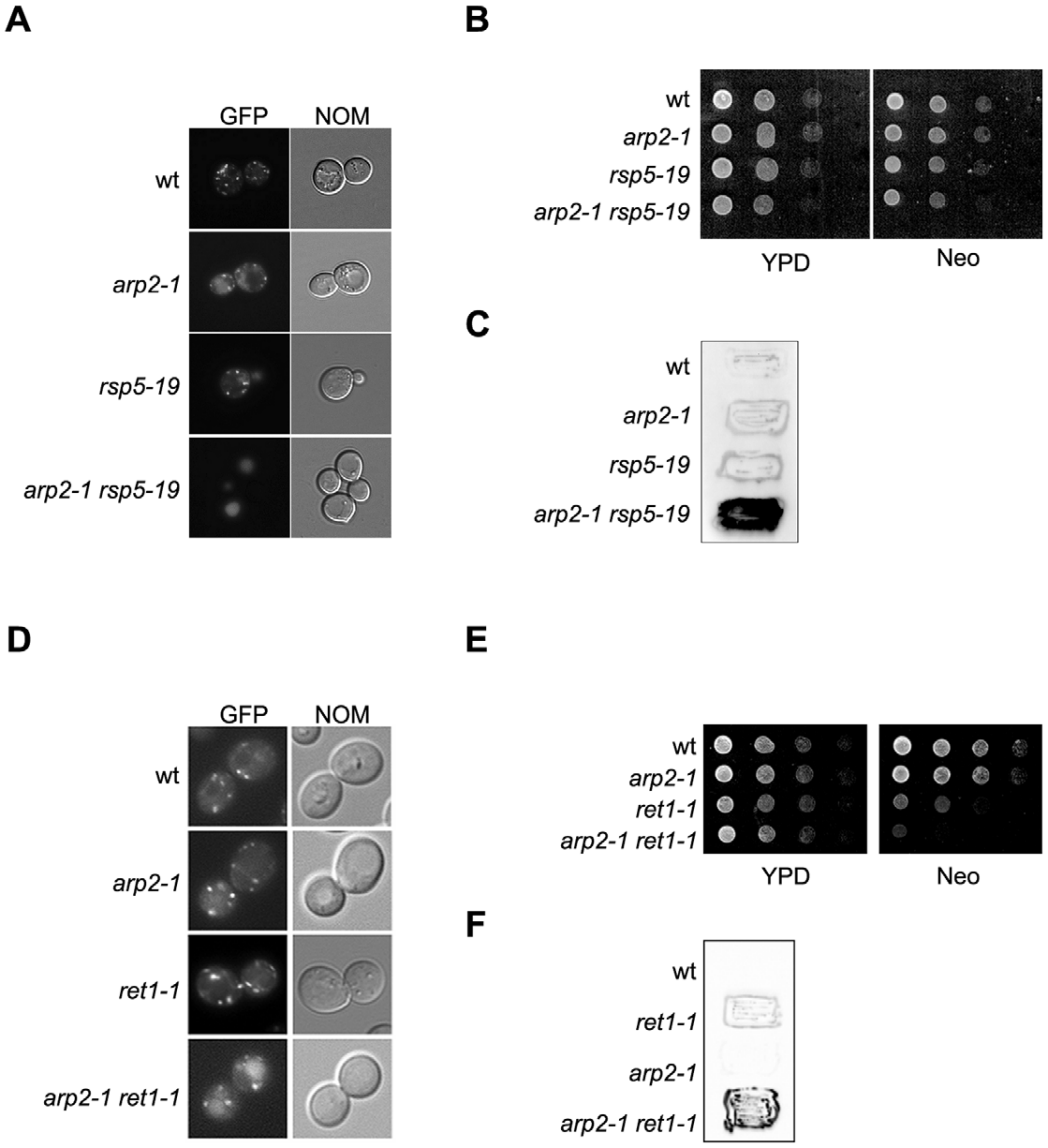
Retrograde trafficking from the Golgi to the ER is impaired in double *arp2-1 rsp5-1* and *arp2-1 ret1-1* mutants. (**A-C**) Analysis of transport from the Golgi to ER in spore clones from cross *arp2-1* × *ret1-1.* (**A**) Defect of GFP-Rer1 trafficking in *arp2-1 rsp5-1* mutants. Spore clones were transformed with plasmid encoding *GFP-RER1*. Transformants were grown and analyzed similarly as spore clones in Figure 4B. (**B**) Double mutant *arp2-1 rsp5-1* is not sensitive to neomycin. Spore clones were serially diluted 1∶10, spotted on YPD or YPD containing 1 mM neomycin (Neo) and grown for 1 day at 28°C. (**C**) Secretion of Kar2 is enhanced in *arp2-1 rsp5-1* mutant. Spore clones were replica-plated onto nitrocellulose filters and grown on solid YPD for 1 day at 28°C. Cells secreting Kar2 were identified by Western blotting with anti-Kar2 antibody. (**D–F**) Analysis of transport from the Golgi to ER in spore clones from cross *arp2-1* × *ret1-1.* (**D**) Defect of GFP-Rer1 trafficking in *arp2-1* × *ret1-1* mutant. The spore clones were transformed with plasmid encoding *GFP-RER1*. Transformants were grown and analyzed similarly as spore clones in Figure 4B. (**E**) Double mutant *arp2-1 ret1-1* is sensitive to neomycin. The sensitivity to neomycin was tested as in B. (**F**) Secretion of Kar2p is enhanced in *arp2-1 ret1-1* mutant. The secretion was assayed as in C.

The accumulation of Sec27 protein in polyubiquitinated form, observed in *rsp5-1* mutant, might change COPI function and in consequence could cause the defect in the COPI trafficking which is additive to the defect caused by *ret1-1* mutation. If this is true, overexpression of *SEC27* should reduce the defect in the Golgi-to-ER trafficking in the *ret1-1 rsp5-1* mutant. To validate this idea we tested if overexpression of *SEC27* is able to attenuate accumulation of GFP-Rer1 in vacuole in the *ret1-1 rsp5-1* mutant. The double *ret1-1 rsp5-1* mutant was transformed with empty plasmid or plasmid overexpressing *SEC27* (Figure 5B). Unexpectedly the overproduction of Sec27 caused fragmentation of vacuoles. The GFP-Rer1 was still in small vacuoles, but some bright punctuate structures were also visible (Figure 5B).

**Figure 8:**
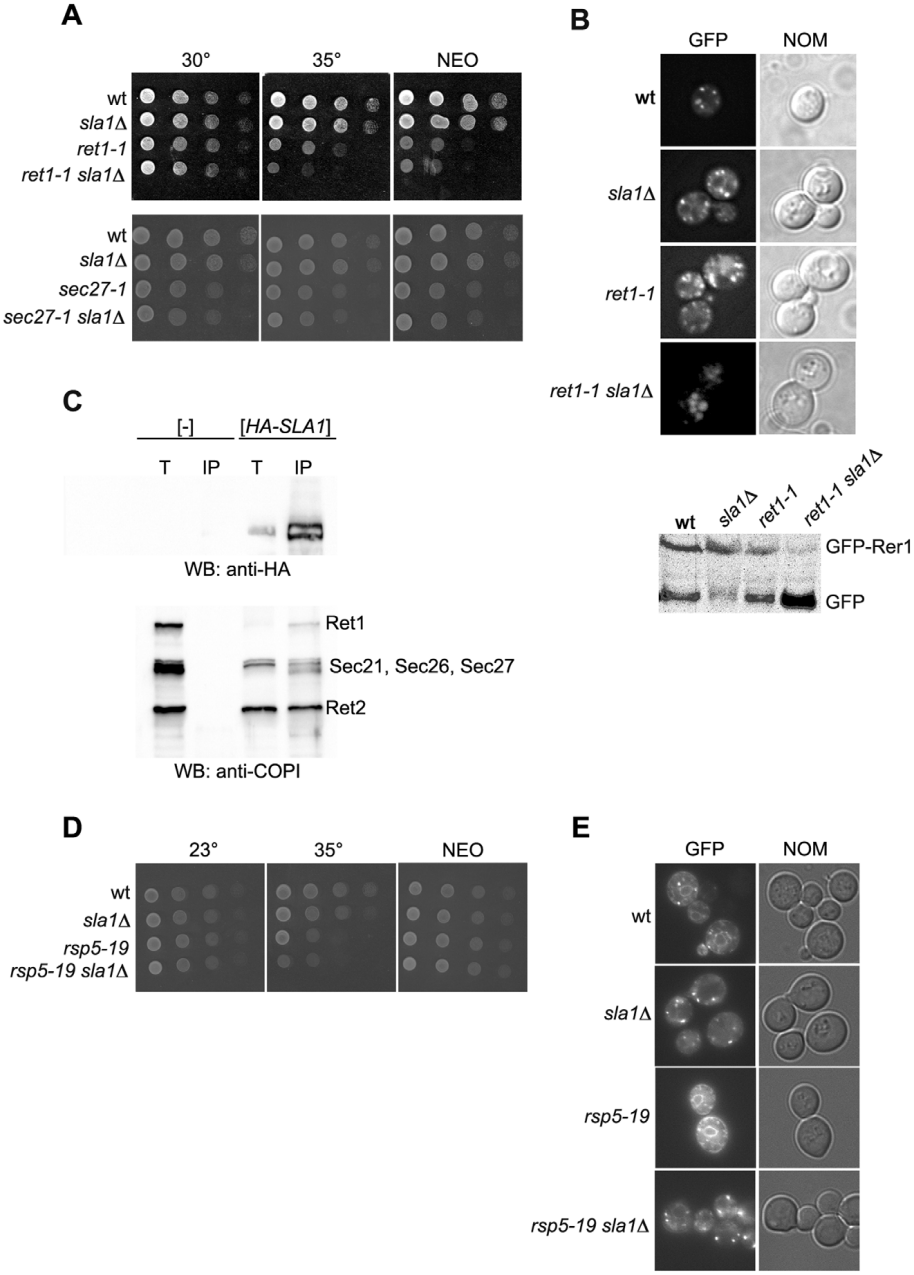
Retrograde trafficking from Golgi to ER is impaired in double *ret1-1 sla1Δ* mutant, but not in *rsp5-19 sla1Δ*. (**A**) Negative genetic interaction between *ret1-1* and *sla1Δ* and no such a genetic interaction between *sec27-1* and *sla1Δ* mutations. Serial 1∶10 dilutions of spore clones from crosses *ret1-1* × *sla1Δ* (JK82-4B × *sla1Δ*) and *sec27-1* × *sla1Δ* (JK84-3C × *sla1Δ*) were spotted on YPD medium or YPD containing 1 mM neomycin (NEO) and incubated for 2 or 3 days at indicated temperatures. (**B**) The spore clones from crosses *ret1-1* × *sla1Δ* were transformed with plasmid expressing GFP-Rer1 and localization of GFP was monitored by fluorescence. (**C**) Sla1 is in complex with COPI proteins. The *sla1Δ* mutant was transformed with empty vector ([-]) or with plasmid expressing *HA-SLA1*. Protein extracts were prepared from transformants and HA-Sla1 was immunoprecipitated with anti-HA antibody. The total protein extracts (T) and immunprecipitated materials (IP) were analyzed by Western blotting with anti-HA and anti-COPI antibody. (**D**) *rsp5-19* and *sla1Δ* mutations have no additive effect on GFP-Rer1 localization. The spore clones from crosses *rsp5-19* × *sla1Δ* were transformed with plasmid expressing GFP-Rer1 and localization of GFP was monitored by fluorescence.

### Overexpression of Ubiquitin Suppresses Some Defects of *ret1-1 rsp5-1* Mutant

To test if a lack of Rsp5-dependent ubiquitination is a reason for the observed interaction between *ret1-1* and *rsp5-1* we checked if overexpression of ubiquitin suppresses the growth defect at 35.5°C and GFP-Rer1 accumulation in the vacuole of the double *ret1-1 rsp5-1* mutant. Mutated variants of ubiquitin with only a single lysine, 48 (K48) or 63 (K63) present, were also tested. The *ret1-1 rsp5-1* mutant grew better at the nonpermissive temperature when transformed with a multicopy plasmid encoding ubiquitin but even better when the K63 variant was used (Figure 6A). Overexpression of native ubiquitin changed the GFP-Rer1 localization from vacuoles to numerous punctate structures (Figure 6B), but expression of the only K48 or only K63 ubiquitin variants did not abolish the vacuolar accumulation of GFP-Rer1 (Figure 6B). The level of free GFP accumulated in the vacuole in the *ret1-1 rsp5-1* mutant was tested by Western blotting. In this mutant as much as 93% of total GFP signal derives from free GFP, indicating an almost exclusive vacuolar localization (and cleavage) of GFP-Rer1. Overexpression of wild type ubiquitin decreased the vacuolar cleavage of GFP-Rer1 to about 62% (Figure 6C). Overexpression of alleles encoding ubiquitin variants showed that only K48 or only K63 had a minor effect on GFP-Rer1 integrity (85% or 78% free GFP). These results suggest that ubiquitination regulates the trafficking of GFP-Rer1 to the vacuole and that formation of differently coupled ubiquitin chains (via both K48 and K63) is important.

Ubiquitination of proteins is a signal for their sorting in endocytic or MVB pathways [16]. Fusion of ubiquitin to these proteins results in their proper sorting in mutants defective in their ubiquitination [31]. In contrast there is no evidence that sorting of protein to COPI vesicles requires their ubiquitination. However, Rer1p has been shown to be ubiquitinated [8], so it is possible that its ubiquitination is a signal for sorting. Additionally, if Rer1 is a substrate for Rsp5p it is easy to explain why overexpression of ubiquitin prevents vacuolar accumulation of GFP-Rer1 in *ret1-1 rsp5-1* mutant. Moreover, this also explains a defect in GFP-Rer1 trafficking in *ret1-1 rsp5-1* mutant as a result of additive effect – impaired function of COPI complex caused by *ret1-1* mutation and lack of GFP-Rer1 ubiquitination due to *rsp5-1* mutation.

To test this presumption we addressed two questions: (1) Is GFP-Rer1 ubiquitinated in Rsp5-dependent manner? (2) Is covalent attachment of ubiquitin to GFP-Rer1 sufficient to prevent its accumulation in a vacuole in the *ret1-1 rsp5-1* mutant? First we checked if Rsp5 is responsible for Rer1 ubiquitination. Wild type and *rsp5-1* mutants strains were transformed with a plasmid expressing His-tagged ubiquitin from the *CUP1* promoter and with a plasmid expressing *GFP-RER1*. Additionally as a control wild type strain was transformed with plasmid expressing His-tagged ubiquitin and with empty vector. Transformants were grown to mid-exponential phase and half of the culture was shifted to 37°C for 1 hour. Next, protein extracts were prepared from cultures grown at both temperatures and His_6_-Ubi was pulled down on Ni-NTA beads. The total protein extracts and the material bound to the beads were analyzed by Western blotting with anti-GFP antibody. As shown in Figure 6D, the single band probably corresponding to monoubiquitinated GFP-Rer1p was detected in extracts from cells expressing *GFP-RER1*, which grew at 30°C and at 37°C regardless of the tested strain. This result suggests that vacuolar localization of GFP-Rer1p in *ret1-1 rsp5-1* mutant is not a result of deficiency in ubiquitination of Rer1p caused by *rsp5-1* mutation.

Next we tested if fusion of ubiquitin to the GFP-Rer1 protein changes its localization. Plasmid encoding Ub-GFP-Rer1 was introduced into *rer1-1 rsp5-1* mutant cells. The localization of Ub-GFP-Rer1 was mostly vacuolar and similar to the localization of GFP-Rer1 (Figure 6D). The observed difference was in the intensity of fluorescence, the signal was stronger for a Ub-GFP-Rer1. Thus ubiquitination of the cargo protein (GFP-Rer1) seems not be important for its proper sorting at the Golgi.

### Rsp5 may Influence Retrograde Golgi-to-ER Trafficking via the Actin Cytoskeleton

Several types of actin and actin-related proteins are found on Golgi membranes, including the GTPase Cdc42p which modulates actin cytoskeleton formation via the actin nucleating complex Arp2/3 interacts with Sec21p (γCOP) [3]. The *rsp5* mutations show a genetic interaction with the *arp2-1* mutation (*ARP2* encodes a subunit of the Arp2/3 complex), and with mutations in the *PAN1* gene or with deletions of *LAS17* (*PAN1* and *LAS17* encode Arp2/3 complex activators) [17], [32], [33]. Therefore, we asked the question if Rsp5p acts in retrograde trafficking indirectly by influencing formation of the actin cytoskeleton. To test this hypothesis we first monitored the trafficking from Golgi-to-ER in *arp2-1* mutant. As shown in Figure 7A *arp2-1* mutant alone does not have defect in trafficking of GFP-Rer1, is not sensitive to neomycin and does not secrete Kar2p as *rsp5-1* cells do. The effect of both *arp2-1* and *rsp5-19* was also monitored. As shown in Figure 7A in double *arp2-1 rsp5-19* mutant the GFP-Rer1 fusion was accumulated in vacuole. We did not observe an enhanced sensitivity to neomycin compared to wild type or to single mutants (Figure 7B), but Kar2p was secreted in the double mutant (Figure 7C). To further support the hypothesis that Rsp5p may influence Golgi-to-ER trafficking by regulating actin cytoskeleton organization we also tested genetic interaction between *arp2-1* and *ret1-1*. The double *arp2-1 ret1-1* mutant exhibited the same phenotypes as *ret1-1 rsp5-1*. It accumulated GFP-Rer1 in the vacuole (Figure 7D), was more sensitive to neomycin (Figure 7E) and secreted Kar2p (Figure 7F). Together these results shows that Arp2p and Rsp5p are important for the transport form the Golgi to the ER and support the hypothesis that Rsp5p influences trafficking from Golgi-to-ER indirectly by regulation of actin cytoskeleton dynamics.

### Sla1, an Actin Cytoskeleton Protein, is Important for the Golgi-to-ER Trafficking

If our hypothesis that Rsp5 influences retrograde Golgi-to-ER trafficking by regulation of actin cytoskeleton dynamics is correct we should be able to find an actin cytoskeleton protein which is a substrate for Rsp5 and is necessary in retrograde trafficking. The mutation in a gene encoding such a protein should also have negative genetic interaction with *ret1-1* mutation. We tested genetic interaction between *sla1Δ, rvs167Δ*, *lsb1Δ*, *lsb2Δ* mutations and *ret1-1*. The additive growth defect was observed between mutations *sla1Δ* and *ret1-1* (Figure 8A). The double *sla1Δ ret1-1* mutant accumulated GFP-Rer1 in a vacuole (Figure 8B) and was more sensitive to neomycin (Figure 8A) compared to the single mutants *sla1Δ* and *ret1-1.* Thus, *sla1Δ* has the same impact on retrograde trafficking form Golgi-to-ER as *rsp5-1*. If Rsp5 participates together with Sla1 in the investigated trafficking we expected that the double mutant *sla1Δ rsp5* has the same phenotypes as each of the single mutants in regard to the GFP-Rer1 localization, neomycin sensitivity and Kar2 secretion. Indeed in the double *sla1Δ rsp5-19* mutant strain there were no changes in GFP-Rer1 localization, Kar2 secretion or neomycin sensitivity compared to single mutants *sla1Δ* or *rsp5-19* (Figure 8D–E). Moreover, HA-Sla1 was co-immunoprecipitated with COPI subunits as was Rsp5 (Figure 8C). Together this results support the hypothesis that Rsp5 might participate in retrograde trafficking from the Golgi-to-ER by its participation in regulation of actin cytoskeleton.

**Table 1:** *S. cerevisiae* strains used in this study.

Strain	Genotype	Source
MHY500	*MAT* **a** *his3Δ-200 leu2-3, 112 ura3–52 lys2–801 trp1–1*	[52]
MHY623	*MATα doa4-Δ1::LEU2 his3Δ-200 leu2-3, 112 ura3–52 lys2–801 trp1–1*	[53]
FW1808	MATα *rsp5-1 his4-912 δR5 lys2-128Δ ura3-52*	F. Winston
RH359-7D	*MAT* **a** *sec27-1 his4 ura3 leu2 bar1*	Laboratory collection
RH3042	*MAT* **a** *ret1-1 his4 ura3 leu2 trp1*	Laboratory collection
RH2948	*MATα his1*	Laboratory collection
PC4	*MATα rsp5-w1 his3Δ-200 leu2-3, 112 ura3–52 lys2–801 trp1–1*	P. Chołbiński
PC7	*MATα rsp5-19 his3Δ-200 leu2-3, 112 ura3–52 lys2–801 trp1–1*	P. Chołbiński
YMW82	*MAT* ***a*** * ade2-101 his3Δ-200 leu2-Δ1 lys2-801 trp1-Δ63 ura3-52 arp2-1*	[54]
KJK39	*MAT* **a** *met15-Δ ura3-Δ his3-Δ leu2-Δ SEC28::kanMX*	OpenBiosystems
KJK74	*MATα doa4-Δ1::LEU2 his3Δ-200 leu2-3, 112 ura3–52 lys2–801 trp1–1 bre5::kanMX*	This study
KJK76	*MATα doa4-Δ1::LEU2 his3Δ-200 leu2-3, 112 ura3–52 lys2–801 trp1–1 HA–rsp5-1*	This study
KJK82	*MATα doa4-Δ1::LEU2 his3Δ-200 leu2-3, 112 ura3–52 lys2–801 trp1–1 bre5::kanMX HA-rsp5-1*	This study
JK39-2A	*MATα ade2 ura3 his3-Δ200 lys2 leu- trp-*	Spore clones from cross YMW82× T82-14C [30]
JK39-2B	*MATα ade2 ura3 lys2 trp-*	[30]
JK39-2C	*MAT* ***a*** * ade2 ura3 lys2 leu- trp-*	[30]
JK39-2D	*MAT* ***a*** * ade2 MOD5 SUP11 ura3 his3-Δ200 lys2 leu-*	[30]
JK82-2A	*MAT* ***a*** * his4 ura3 ret1-1*	Spore clone from cross FW1808× RH3042
JK82-2B	*MATα leu2 leu2 his4 ura3 rsp5-1*	Spore clone from cross FW1808× RH3042
JK82-2C	*MATα lys2 his4 ura3*	Spore clone from cross FW1808× RH3042
JK82-2D	*MAT* ***a*** * lys2 leu2 his4 ura3 ret1-1 rsp5-1*	Spore clone from cross FW1808× RH3042
JK84-3A	*MAT* ***a*** * lys2 leu2 his4 ura3 sec27-1 rsp5-1*	Spore clone from cross FW1808× RH359-7D
JK84-3B	*MATα his4 ura3*	Spore clone from cross FW1808× RH359
JK84-3C	*MATα his4 ura3 sec27-1*	Spore clone from cross FW1808× RH359
JK84-3D	*MAT* ***a*** * lys2 leu2 his4 ura3 rsp5-1*	Spore clone from cross FW1808× RH359
JK107-1D	*MAT* **a** *rsp5-w1 his3Δ-200 leu2-3, 112 ura3–52 lys2–801 trp1–1*	Spore clone from cross MHY500× PC4
JK139-1A	*MATα ade2-1 lys2 leu2 ura3 trp1 his3 his4 arp2-1*	Spore clone from cross JK82-2A × YMW82
JK139-1B	*MAT* ***a*** * lys2 leu2 ura3*	Spore clone from cross JK82-2A × YMW82
JK139-1C	*MATα lys2 leu2 ura3 trp1 ret1-1 arp2-1*	Spore clone from cross JK82-2A × YMW82
JK139-1D	*MAT* ***a*** * ade2-1 lys2 leu2 ura3 his3 his4 ret1-1*	Spore clone from cross JK82-2A × YMW82
JK140-5A	*MATa leu2 trp1-1 ura3-52 lys2 his3 bre5::kanMX*	Laboratory collection
KJK135	*MAT* ***a*** * lys2 leu2 his4 ura3 trp1 ::kanMX ret1-1 rsp5-1*	Derivative of JK82-2D
JK164-4A	*MATα sla1::kanMX ret1-1 lys2 his ura3 leu2*	spore clone from cross JK82-4B × sla1Δ (OpenBiosystems)
JK164-4B	*MATα met15 his ura3 leu2*	spore clone from cross JK82-4B × sla1Δ (OpenBiosystems)
JK164-4C	*MAT* ***a*** * ret1-1 met15 lys2 his ura3 leu2*	spore clone from cross JK82-4B × sla1Δ (OpenBiosystems)
JK164-4D	*MAT* ***a*** * sla1::kanMX ura3 leu2*	spore clone from cross JK82-4B × sla1Δ (OpenBiosystems)
JK187-1A	*MAT* ***a*** * ura3 leu2 his3*	spore clone from cross PC7× sla1Δ (OpenBiosystems)
JK187-1B	*MATα ura3 leu2 his3 met15 lys2 trp1 sla1::kanMX rsp5-19*	spore clone from cross PC7× sla1Δ (OpenBiosystems)
JK187-1C	*MAT* ***a*** * ura3 leu2 his3 met15 rsp5-19*	spore clone from cross PC7× sla1Δ (OpenBiosystems)
JK187-1D	*MATα ura3 leu2 his3 lys2 trp1 sla1::kanMX*	spore clone from cross PC7× sla1Δ (OpenBiosystems)

## Discussion

In this work, we present evidence that Rsp5p ubiquitin ligase, besides its well documented role in the entry of proteins into endocytic or MVB vesicles, also regulates the trafficking in the early secretory pathway between the Golgi apparatus and the ER. This is in addition to the recently published data that Rsp5p can, at least *in vitro,* ubiquitinate Sec23p, a subunit of COPII coat [21]. Our data provide different lines of evidence indicating that Rsp5p regulates retrograde trafficking to ER. First, there is genetic interaction between the *rsp5-1* mutation and *ret1-1* or *sec28Δ* both affecting Golgi-to-ER transport. Second, the double mutant *ret1-1 rsp5-1* shows enhanced phenotypes characteristic for mutants with defective Golgi-to-ER trafficking (accumulation of GFP-Rer1 in the vacuole, secretion of Kar2p) and added sensitivity to neomycin. It can thus be concluded that Rsp5p regulates COPI operation at the Golgi. Cooperation of COPI and Rsp5p in MVB formation cannot be completely excluded, but some findings argue against it. The level of CPY secretion is not increased in the *ret1-1 rsp5-1* double mutant relatively to that in *ret1-1*. CPY sorting is regarded as an indicator of endosomal function. Partial sorting defects, like in the class E *vps* mutant *vps4*, cause a substantial fraction of CPY to be secreted [34]. The lack of an effect of the *rsp5-1* mutation on endosomal sorting is in agreement with the results of Katzman and co-workers who found that the *rsp5-1* mutant did not secrete CPY [35]. Thus, the endosomal function seems not to be perturbed by the *rsp5-1* mutation. Second, Rer1p is a transmembrane protein and has to be sorted in the MVB. Cleaved GFP from the GFP-Rer1 fusion was found in the vacuolar lumen in the *ret1-1 rsp5-1* mutant suggesting unperturbed trafficking to the vacuole. This is in agreement with the suggestion that ubiquitination by Rsp5p ligase is required at this stage of trafficking for selective cargo recognition rather than for MVB formation. Moreover, the mutant used here, *rsp5-1*, was described earlier as showing no defect in the ubiquitination of carboxypeptidase S precursor (pCPS) [35]. Also the defect in Sna3-GFP fusion protein sorting into the vacuole caused by the *rsp5-1* mutation is not enhanced by *ret1-1*. Another possibility is that the observed genetic interaction is a result of an additive defect in anterograde trafficking caused by *ret1-1* and *rsp5-1*. The *ret1-1* mutation inhibits the transport of Gas1p, a glycosylphosphatidylinositol (GPI)-anchored protein, and other GPI-anchored proteins [36], while the transport of CPY [2] or invertase proceed with wild type kinetics [36]. However, strong inhibition of Gas1 transport is also observed in the *sec21-1* mutant, but there is no genetic interaction between *sec21-1* and *rsp5-1* (J.K unpublished data). Moreover, *ret1-1 rsp5-1* double mutant secretes invertase normally. So it seems that the defect in transport of GPI-anchored proteins and invertase is not the reason of the observed growth defect of the *ret1-1 rsp5-1* mutant.

The idea that Rsp5p regulates, together with COPI, trafficking at the early Golgi is further supported by the finding that Bsd2p, an adaptor protein for Rsp5p, competes with Rer1p for transmembrane proteins [37]. In addition we were able to co-immunoprecipitate COPI subunits together with HA-Rsp5.

By what mechanism does Rsp5p influence the retrograde trafficking to the ER? There are at least four possibilities: (1) Rsp5 could ubiquitinate cargo proteins to target them into COPI vesicles; (2) Rsp5 might regulate Sec27 function; (3) Rsp5 might ubiquitinate COPI proteins other than Sec27; (4) Rsp5 regulates the Golgi-to-ER trafficking indirectly, for example by influencing proteins regulating later steps in COPI vesicle biogenesis, its fission, transport of fusion.

The first explanation is unlikely because even though the Rer1p receptor is known to be ubiquitinated [8], the ubiquitinated form of Rer1 is detected in *rsp5-1* mutant and fusion of ubiquitin to GFP-Rer1 does not changes its localization in *ret1-1 rsp5-1* mutant. Additionally, the additive sensitivity to neomycin of the *rsp5-1* mutation with *ret1-1* supports the idea of a general role of Rsp5 in the regulation of coatomer function.

The second explanation would be consistent with the genetic data if *sec27-1* is defective in the Rsp5-dependent regulation. In this case there would not be a synthetic interaction between the two alleles. The presence of a genetic interaction between mutations in two of the three genes encoding subcomplex B subunits (*ret1-1* and *sec28Δ* with *rsp5-1*, would be consistent as well. This interpretation is also supported by the finding that there is a strong genetic interaction between mutated alleles of *RET1* and *SEC27* genes, similar to the *ret1-1 rsp5-1* one ([38], our unpublished observation). On the other hand, the compromised growth of the *ret1-1 rsp5-1* mutant can be due to a defect of Kar2p transport and probably other -HDEL motif-containing proteins from the Golgi to the ER. Saturation of this system inhibits growth [39]. No Kar2p secretion is observed in the *sec27-1 rsp5-1* mutant, which correlates with the lack of an additive growth defect of the *sec27-1* and *rsp5-1* mutations. Still, the double *sec27-1 rsp5-1* mutant has an additive defect in GFP-Rer1 retrieval to the ER compared with the single mutants, suggesting that Rsp5p regulates retrograde transport.

The obtained results suggest a role of Rsp5p in changing the mode of Sec27p action in the COPI complex. In the double *ret1-1 rsp5-1* mutant the changes in Sec27p operation caused by the *rsp5-1* mutation (accumulation of polyubiquitinated Sec27p) would have an additive effect with that caused by *ret1-1* and in consequence would enhance the defect in retrograde trafficking. The experiment designed to test this hypothesis – testing the effect of overexpression of *SEC27* on GFP-Rer1 trafficking in *ret1-1 rsp5-1* mutant, did not give an answer. The suppression of vacuolar accumulation of GFP-Rer1 and of the temperature sensitivity of *ret1-1 rsp5-1* by overexpression of ubiquitin suggests that a process defective in the double mutant relies on ubiquitination. Further studies are needed to establish the type of ubiquitination affected. Testing GFP-Rer1 localization in strains defective in the formation of specific ubiquitin chains (SUB strains) failed to provide an answer – in all these strains the localization of GFP-Rer1 was unperturbed (J.K., unpublished data). The decreased ability of ubiquitin with only K48 or only K63 to suppress the *ret1-1 rsp5-1* mutant defects suggests the action of more than one ligase similarly as in the case of Rbp1p [40]. This raises the possibility that Sec27p is ubiquitinated by Rsp5 even though ubiquitinated Sec27p is still detected in the *bre5Δ rsp5-1* mutant and can explain why we observe changes in ubiquitination pattern in the *rsp5-1* mutant after shift to nonpermissive temperature.

The third possibility is that Rsp5 influences ubiquitination of other COPI subunits or other protein regulating the formation of COPI vesicles, because subunits Ret1p, Ret3p, Sec21p, Sec26p and Sec28p were also found to be ubiquitinated [8]. Also the regulatory effect of Rsp5p on COPI function might be connected with the ability of WD40 domains of Sec27 or Ret1 to bind ubiquitin [41].

The fourth possibility is that Rsp5 affects trafficking from Golgi to ER by influencing formation of the actin cytoskeleton. This hypothesis is supported by our finding that the *arp2-1* mutation has similar effect on traffic as *rsp5-1* and that *arp2-1 ret1-1* mutant accumulates GFP-Rer1 in the vacuole, is more sensitive to neomycin and has enhanced secretion of Kar2p. This hypothesis is also supported by the finding that Rvs167p, a protein involved in actin cytoskeleton dynamics and a substrate for Rsp5p [42], has been found in complexes with Sec21p [43] that Arp2 is required for efficient retrograde traffic, as is Sla1, a multi-domain protein and a substrate for Rsp5 [42]. The double *ret1-1 sla1Δ* mutant has similar phenotypes as *ret1-1 rsp5-1* with respect to GFP-Rer1 trafficking, Kar2 secretion and neomycin sensitivity. Moreover, the double *sla1Δ rsp5-19* mutant has no enhanced defect in the Golgi-to-ER trafficking compared to the single mutants. The interpretation of these genetic relationships is that Sla1 and Rsp5 regulate retrograde trafficking through the same pathway. Further work should clarify the molecular mechanism by which Rsp5p participates in COPI-dependent Golgi-to-ER trafficking.

## Materials and Methods

### Strains, Media and Growth Conditions

The *Escherichia coli* strain DH5αF’ [*F’ supE44 ΔlacU169 (Φ80 lacZΔM15) hsdR17 recA1 endA1 gyrA96 thi-1 relA1*] was used for cloning and plasmid propagation. The plasmids used in this study were: pSKY5/RER1-0 (*GFP-RER1*, *CEN*, *URA3*) [44], YEp96, pTer78 and pTer79 (P*_CUP1_-myc-UBI*, -*ubi K48* or -*ubi K63*, where all other lysines are replaced with arginines) (gift from M.J. Ellison), P*_CUP1_-HIS_6_-UBI*, [45], YCp33-HA-RSP5 [24], and YCpJYS-22 (*DOA4, CEN, URA3*) [46]. Plasmid pRS424 P_ADH1_SEC27 was created by amplification of *SEC27* gene from plasmid BG1805-SEC27 (Open Biosystems) with primers having additional overhangs allowing for cloning of PCR product into PstI SalI restriction sites of pRS424 P_ADH1._

To obtain the gene fusion *UBI-GFP-RER1* plasmid pSK5 was digested with NotI enzyme and religated to obtain plasmid pSK5ΔNotI without *TDH3* promoter sequence. Next *TDH3-UBI* fusion was constructed by fusion-PCR. Plasmid Yep96 was used as a template to amplify ubiquitin gene and to add 21 bp overhang of *TDH3* promotor at 5′ end and NotI site in the 3′ end. *TDH3* promotor was amplify on pSK5 plasmid and 19 bp corresponding to ubiquitin gene was added to 3′ end. Next fusion-PCR was performed. The *TDH3-UBI* fragment was ligated into NotI site of pSK5ΔNotI.

The *S. cerevisiae* strains used are listed in Table 1. Experiments done on spore clones were always performed on two independent tetra type tetrads, the representative results done on one of them are shown. Yeast growth followed standard procedures [47]. YPD (1% yeast extract, 1% peptone, 2% glucose), synthetic drop out (SC -ura, SC -trp or SC -trp -ura), SC +5′ fluorouracil (5′ FOA) and synthetic minimal medium (SM) were used [47]. Yeast strains were transformed as in [48]. The KJK74 strain was obtained by deletion of the *BRE5* gene in MHY623 using a PCR product according to the method of [49]. KJK76 and KJK82 were obtained by *RSP5* allele replacement in MHY623 and KJK74, respectively. *YIP-HA-rsp5-1* was linearized with PstI. Integrants were selected on SC-ura dropout plates and then replica-plated on 5′ FOA plates and on YPD incubated at 37°C to select for cells that had lost the *URA3* marker and were temperature sensitive. The allele replacement and the presence of the HA tag was confirmed by PCR.

### Total Protein Extracts, Immunoprecipitations and Western Blot Analysis

Extracellular Kar2p secretion was analyzed as described in [50]. Protein extracts to monitor the GFP-Rer1 or Sna3-GFP processing in the vacuole were prepared as described in [32]. The immunoprecipitation was done as in [32] The rabbit polyclonal antibodies used in the study were: anti-Kar2 (from M. Rose), anti-CPY [51], anti-COPI (from A. Spang) and anti-Sec27 (from F. Letourneur). Mouse monoclonal antibodies were: anti-GFP (Roche), anti-HA (Babco) and anti-PGK (Invitrogen). Secondary anti-mouse or anti-rabbit HRP-conjugated antibodies were from DACO. The Westerns were developed with an enhanced chemiluminescence kit from Millipore. The intensity of bands was calculated with ImageQuant 5.2 software.

### His_6_-Ubi Pull Down

The pull down of His_6_-tagged ubiquitinated proteins was performed two times as described in [28] with some modifications. For testing of Sec27p ubiquitination strains MHY623, KJK74, KJK76 and KJK82 were used and for GFP-Rer1 ubiquitination strains MHY501 and PC10 All above strains were transformed with plasmid P*_CUP1_HIS_6_-UBI* and MHY501 and PC10 additionally with plasmid pSKY5. Transformants were grown to mid-exponential phase at 30°C. Next, Cu^2+^ was added to a final concentration of 100 µM. To monitor Sec27p ubiquitination half of each culture was incubated at 30°C and half at 37°C for 4 hours. To monitor GFP-Rer1 ubiquitination cultures were first incubated with Cu^2+^ at 30°C for 2 hours and half of the culture was shifted to 37°C for 1 hour. The same number of cells from each culture was harvested and disrupted with glass beads in lysis buffer (100 mM NaPO_i_ pH 8.0 10 mM Tris pH 8, 6 M guanidine, 5 mM imidazole, 10 mM mercaptoethanol, 0.1% Triton X-100). The lysate was incubated with Ni-NTA beads for 2 hours and washed with lysis buffer and with washing buffer (100 mM NaPO_i_ pH 6.4 10 mM Tris pH 6.4, 8 M urea, 10 mM mercaptoethanol, 0.1% Triton X-100). Fraction bound to beads was eluted with sample buffer. All buffers except the latter were supplemented with protease and proteasome inhibitors.

### Invertase Activity Assay

Invertase activity was assayed as in [52]. The activity of invertase was assayed twice from three independent cultures for each strain.

### Fluorescence Microscopy

For GFP fluorescence yeast were grown to the logarithmic phase in indicated medium at indicated temperature. Staining with CellTracker™ Blue CMAC (7-amino-4-chloromethylcoumarin) was performed as in [28]. Cells were mounted on a slide and were viewed with an Eclipse fluorescence microscope (Nikon) equipped with an ORCA (Nikon) camera. Images were collected using Lucia General 5.1 software (Laboratory Imaging Ltd.). The percentage of cells accumulating GFP-Rer1 fusion in the vacuole was counted for 150–250 cells.
